# Educator perspectives on costs and cost-conscious decision-making in health professions education: a Q-Method study

**DOI:** 10.1007/s10459-025-10463-2

**Published:** 2025-08-06

**Authors:** Jennifer Yaros, Mirjam Oude Egbrink, Benedikt Langenberg, Silvia Evers, Aggie Paulus

**Affiliations:** 1 Department of Health Services Research (HSR), Maastricht University, Faculty of Health Medicine and Life Sciences, School of Health Professions Education (SHE), Maastricht, The Netherlands; 2 Department of Physiology, Maastricht University, Faculty of Health Medicine and Life Sciences, School of Health Professions Education (SHE), Maastricht, The Netherlands; 3 Department of Methodology and Statistics, Maastricht University, Faculty of Health Medicine and Life Sciences, Care and Public Health Research Institute (CAPHRI), Maastricht, The Netherlands; 4 Department of Health Services Research, Maastricht University, Faculty of Health Medicine and Life Sciences, Care and Public Health Research Institute (CAPHRI), Maastricht, The Netherlands; 5 Trimbos Institute, Center for Economic Evaluation and Machine Learning, Netherlands Institute of Mental Health and Addiction, Utrecht, The Netherlands; 6 Department of Health Services Research, Maastricht University, Faculty of Health Medicine and Life Sciences, School of Health Professions Education (SHE), and Care and Public Health Research Institute (CAPHRI), Maastricht, The Netherlands; 7 Department of Health Service Research, Maastricht University, Maastricht, The Netherlands

**Keywords:** Costs, Cost-conscious decision-making, Health professions education, Q-Method

## Abstract

Intensification of resource scarcity in health professions education (HPE) requires careful consideration of educational costs. Educators play a significant role in this process, as they make resource allocation decisions when designing, developing, organizing and overseeing education, yet little is known about how educators view the idea of incorporating information on educational costs into decision-making. Therefore, this study maps educator views on cost-conscious decision-making in HPE. Using Q-Methodology, we explored how HPE educators perceive costs and cost-conscious decision-making. Participants ranked 34 statements and engaged in semi-structured interviews. By-person inverted factor analysis with varimax rotation was used to extract factors representing distinct patterns of thinking held by three or more educators. These patterns were interpreted using factor arrays, distinguishing and consensus statements, and interview transcripts, yielding narrative descriptions of unique educator perspectives. Twenty-five educators holding diverse educational responsibilities and 1–29 years of experience were classified into four distinct perspectives. *Unaware Doubters* (n = 3) are unexposed to educational cost considerations and lack motivation for involvement. *Cautious Realists* (n = 8) are personally impacted by resource constraints, yet hesitant about cost-conscious decision-making. *Pragmatic Supporters* (n = 8) hold implicit awareness of resource scarcity but remain open to participation in cost-conscious decision-making. *Empowered Agents* (n = 6) are aware, active and engaged in cost-conscious decision-making, yet seek a unifying vision and transparent communication from leadership. These four distinct educator perspectives on costs and cost-conscious decision-making in education illustrate varying levels of awareness, motivation and engagement, which indicates the need for tailored strategies to improve cost-conscious decision-making in HPE. This information can be used by educational institutes to develop a vision, policy and strategy to involve educators in considerations concerning educational costs in view of the increasing resource scarcity in HPE. Future research should evaluate the effectiveness of different strategies targeted to these perspectives and explore how perspectives vary across settings and contexts.

**Supplementary information:**

The online version contains supplementary material available at 10.1007/s10459-025-10463-2.

## Introduction

Resource scarcity is increasingly impacting health professions education (HPE) (Cleland et al., 2020; Maloney et al., 2019). Attempts to navigate shortages of time and money (APPA, 2013) lead decision-makers to consider the impacts of different educational alternatives, such as utilizing peer teachers (Aamodt et al., 2006; Wagner et al., 2017) or standardized patients (Bosse et al., 2015), implementing a five vs eight station Objective Structured Clinical Examination (Goh et al., 2022), or opting for either online (Alton et al., 2018; Bandla et al., 2012; Young & Greenberg, 2013), blended (Kumpu et al., 2016) or face-to-face (Maloney et al., 2015; Rees & Gillam, 2001) educational components. To optimize these and other resource allocation decisions, it is imperative that we know something about both the quality of education and the costs of these alternative resource uses (Foo et al., 2021). Without this information, we may unconsciously select options which fail to provide the highest quality education or the most reasonable cost.

Within HPE there are many stakeholders responsible for making resource allocation decisions, including educational leaders, administrators, and financial managers. Yet the educators responsible for carrying out educational activities also implicitly impact resource allocation decisions when designing, delivering and overseeing education. In this way, they also influence and hold some responsibility for the costs and quality of education. While we are fairly certain that educators strive to improve the quality of education, to the best of our knowledge little is known about how they consider educational costs.

From the broader literature pertaining to health professions, there have been several investigations examining how providers perceive cost-conscious decision-making (Carroll et al., 2024; Cooke, 2010; Leep Hunderfund et al., 2017; Moleman et al., 2021,2022; Mordang et al., 2020; Stammen et al., 2019; Stammen et al., 2015), but within HPE there is but a single investigation exploring educators’ attitudes towards economic research (Cleland et al., 2022). Here it was shown that educational leaders were hesitant to adopt economic research practices due to economic illiteracy, fear of increased accountability, concerns of autonomy loss, and a strong educator identity (Cleland et al., 2022). However, this evidence was derived from a small-scale, qualitative study that only included the perspective of educational leaders. Therefore, the perspectives of all other HPE educators remains heretofore unexplored. Furthermore, this investigation only explored attitudes toward the application of economic evidence in HPE decision-making, not educators’ perceptions towards the incorporation of educational costs into everyday decision-making. For this, we only have the suggestions of experts that HPE educators may be skeptical and lack motivation for cost-conscious decision-making (Walsh, 2013), or that a lack of demand from policymakers may hamper educator engagement in such practices (Levin, 2001). However, none of these presumptions have been substantiated through quantitative nor qualitative evidence. Therefore, we cannot say if educators are conscious of resource scarcity in HPE, what their current level of motivation may be for incorporating costs into educational decisions, nor what it prerequisites may exist to garnering their engagement in cost-conscious decision-making practices. Yet, this information is vital to fostering a cost-conscious mindset in HPE.

To address this knowledge gap, comprehensive insights from educators at different career stages and representing different educational roles and responsibilities are needed. Therefore, this study employs a mixed-methods approach to explore how novice, experienced and expert educators (with 0–6, 6 > < 15, 15 + years of experience) perceive cost-conscious decision-making in HPE. By mapping their responses in diverse perspectives in accordance with the Integrated Change Model of behavioral change (de Vries, 2017), we seek to uncover distinctions in educator awareness, motivation and action with cost-conscious decision-making, and provide a foundation for developing relevant and beneficial strategies to adopt and integrate cost-conscious practices into HPE decision-making. Ultimately, such insights may contribute to a system-wide shift towards efficient resource allocation and ensure economic sustainability and quality in HPE in a resource-constrained environment.

## Aim

The aim of this investigation is to illuminate distinctions in educator perspectives towards costs and the use of cost information in HPE decision-making.

## Research question

The primary research question is: What are educators’ perspectives on taking costs into consideration when making educational decisions?

## Methods

### Setting

This study was conducted at a single setting, Maastricht University, in The Netherlands. Maastricht University was founded upon the system of Problem-Based Learning (Barrows, 1996) (PBL), which is based on the principal that education should be constructive, collaborative, contextual and self-directed. Within the Faculty of Health Medicine and Life Sciences (FHML), the Institute for Education is responsible for the organization of education and sets the educational norm hours (standards) associated with different teaching roles. Teaching roles are divided into three categories: 1) Oversight, 2) Development and Organization, and 3) Delivery of Education. Oversight of education is defined as coordination of courses or programs and includes roles such as course and program coordinators, (vice-)chairs of committees, scientific directors, and so on. Educators in positions of oversight are responsible for distributing educational hours among teaching staff to achieve intended learning goals. Development and organization of education is defined as the design and coordination of small-scale educational components, such as a single topic or task within a course, and includes roles such as members of planning groups, program committees or Board of Examiners. Delivery of education is defined as direct-student contact time and includes such roles as tutors, lecturers, mentors, coaches, trainers, assessors and so forth. At Maastricht University, education is delivered in small-scale learning environments, which necessitates almost all educators to participate in the delivery of education. Furthermore, it is common for all but the most novice educators to hold dual or triple responsibilities. All educators are responsible for working within the limits of educational hours allotted to their position and responsibilities. For the purpose of this study, educator experience is defined in terms of novice (0–6 years), experienced (6 > < 15 years), and expert (15 + years).

### Study design

This investigation was conducted using Q-Methodology (Brown, 1993). Q-Methodology is an exploratory mixed-methods approach that utilizes by-person (inverted) factor analysis to illuminate distinct patterns of thinking about controversial topics (Zabala et al., 2018). In contrast to traditional factor analysis, which analyses variables across a set of participants, by-person factor analysis analyses participants across a set of statements that represent their patterns of thinking (see Exploratory Analysis for additional explanation).This approach suited the aim of this investigation to explore educator perspectives on cost-conscious decision-making in HPE, which is heretofore unexplored, and is not designed to correlate participant characteristics with the outcomes of the analysis. This investigation was carried out in the following stages: defining the concourse, developing the statement set, selecting participants, collecting data, conducting by-person inverted factor analysis, interpreting the data and reporting results.

### Defining the concourse

In Q-Methodology, the concourse is considered a representation of the “universe of opinions” on the topic of investigation and the source from which statements are drawn (Watts & Stenner, 2012). This concourse was defined from scientific literature, editorials, commentaries, and informal conversations with HPE stakeholders that took place while conducting and presenting research on HPE costs (Yaros et al., 2023,2024). These conversations occurred in the workplace at Maastricht University, FHML, School of Health Professions Education and Department of Health Service Research, and at academic conferences, including the Association for Medical Education Europe and Netherlands Society on Medical Education.

Educator participation in cost-conscious decision-making was viewed as a shift in individual, organizational and cultural behavior. Thus, the concourse was structured in accordance with the Integrated Change Model (de Vries, 2017) (I-Change) to reflect three aspects of behavioral change: 1) awareness, including cognizance, knowledge, risk perception and perceived cues; 2) motivation, including attitudes, social support, self-efficacy and intention; and 3) action, including action planning, enactment, skills and barriers. Content analysis was performed to classify and verify that all three aspects of behavioral change were represented in the data and was iteratively discussed by the members of the research team. Once agreement and data saturation were reached, the phase of defining the concourse ended, and the development of the statement set began.

### Developing the statement set

Statements were developed iteratively to represent the diversity of opinions found in the concourse. Initially, 60 statements were created to reflect varying degrees of awareness, motivation and action from educators at different stages of their career and with differing educational responsibilities. This statement set was discussed by the research team to improve clarity and comprehensiveness and thereby reduced to 43 statements. These 43 statements were then pilot tested by seven potential participants: two novice educators with less than five years’ experience in the delivery of education, and two experienced educators with 12–15 years’ experience and three expert educators with greater than 30 years’ experience; the latter two groups have experience in the delivery, development and organization, and oversight of education. Feedback from these respondents influenced the statement set in several ways. First, statements were reworded to improve consistency, understanding and relevance for a broad audience of educators. Second, redundant and tangentially related topics, such as resource scarcity, cost evidence, economists, and economic evaluations, were removed or condensed accordingly, reducing the set to 32 statements. Lastly, missing concepts related to willingness to use costing guidelines and fears of cost considerations overtaking quality considerations, led to the creation of two additional statements, resulting in a final set of 34 statements (Supplemental File).

### Selecting participants

Participants were purposively sampled based on their relevance to this investigation as professional educators employed in the field of HPE. The assumption was that these individuals hold distinct views on the topic of investigation and, therefore, maximize the potential for all major factors of influence to be represented in the data. Educators were eligible for inclusion in this study at any stage of their career: novice (0–6 years), experienced (6 > < 15 years) or expert (15 + years) and if they held responsibilities for delivery, development and organization, or oversight of education at any time in the past three years.

In Q-Methodology, participants are the variables whose subjective opinions are explored to establish patterns of similarities and differences in point of view (Watts & Stenner, 2012). It is therefore generally accepted that a sample size less than the number in the statements set is sufficient to establish commonality in distinct perspectives (Watts & Stenner, 2012). To ensure adequate and balanced representation of educators with various responsibilities at varying phases of their careers, while in keeping with this recommendation, we aimed to include ten novices, ten experienced and ten expert educators.

### Collecting data

**Fig. 1:**
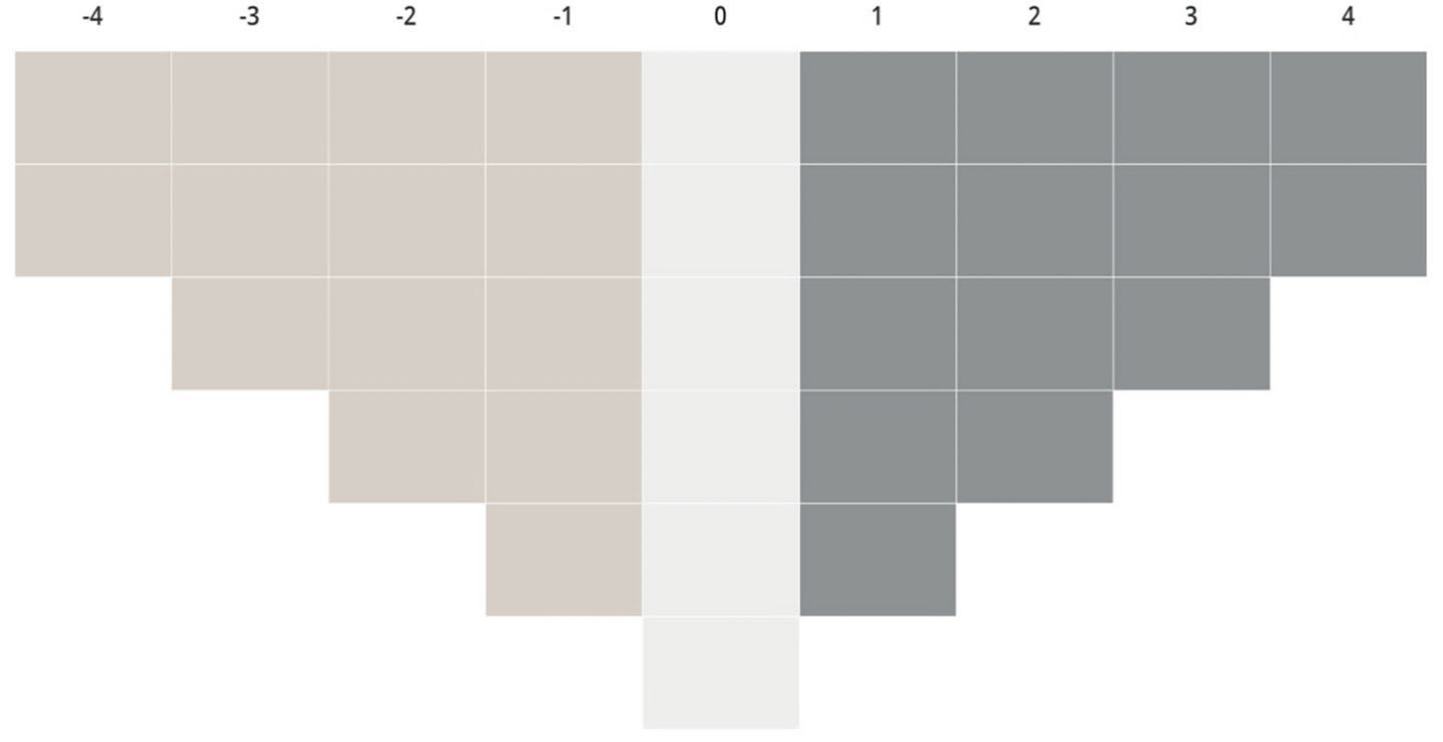
Response grid

Data collection and interviews were conducted one-on-one by the lead researcher (JY) and occurred via one of two means: in-person or online via Q-Method Software (Lutfallah & Buchanan, 2019), based on participant preference. Both formats were pilot tested to ensure instructional clarity and feasibility of task completion within 60 minutes. At the start of each session, participants were presented with the research question and provided with the statement set. They were asked to read through all statements and roughly sort them into three piles: those they strongly agree with, feel neutral towards or strongly disagree with. Then they were asked to rank the statements relative to one another on a nine-point (−4 to + 4) response grid in the shape of an inverted triangle (Fig. 1).

The response grid followed a normal pattern of distribution and allowed for only two statements at either extreme (−4 and + 4). This steeper distribution forced participants to choose the two statements they most strongly agreed with and the two they most strongly disagreed with, mimicking real-world decision-making constraints. In keeping with the recommendations of Brown (1980), this also allowed participants to rank more statements towards the mid-section, reflecting indifference towards a topic in which they were expected to lack familiarity. Ranking of statements continued until participants felt the response grid provided the best possible reflection of their perspective. The completed response grid is known as a participant Q-sort.

Immediately following the completion of the Q-sort, an interview was conducted to explore participants’ reasoning for their choices. During the interview, participants were asked to explain the statements they felt most strongly about, their general understanding of educational costs and cost-conscious decision-making, who they felt should be involved in these considerations and what support is necessary to garner their involvement. In addition, participants were asked if any statements were unclear, missing or if there was any additional information they wish to share (Supplemental File).

### Conducting by-person factor analysis

Data from the Q-sorts was analyzed in KADE open-source software (Banasick, 2019), in consultation with a statistician (BL). This was an iterative process that repeatedly cycled through the following steps. An exploratory analysis of the data was conducted to inform selection of analytical method and to determine the number of factors to retain in the solution (Supplemental File). A by-person inverted factor-analysis was conducted to extract factors that identify distinct patterns of thinking among educators. A varimax rotation of factors was performed to clarify distinctions between these patterns and improve the interpretability of the solution. Rotated factors were interpreted with qualitative data and used to draft narrative descriptions. A sensitivity analysis of factor loading cut-off values was performed to enhance the interpretability of factors and narrative descriptions were updated accordingly.

#### Exploratory analysis

Q-Methodology employes by-person inverted factor analysis to extract factors representative of distinct patterns of thinking among participants. This is a statistical method that inverts traditional factor analysis (Watts & Stenner, 2012). Rather than analyzing variables across a sample of individuals, this approach analyzes individuals across a set of variables (i.e. the statement set) (Akhtar-Danesh, 2018). A correlation matrix is derived from the statement ranking patterns represented in the Q-sorts and thus represents the associations between individuals’ responses. Factors are then extracted from the correlation matrix representing distinct patterns of thinking (i.e. perspectives) observed in the Q-sorts. Participants are associated with these perspectives based on how closely their Q-sort aligns with a factor’s defining features, measured by factor loading values.

KADE software (Banasick, 2019) offers two means of by-person factor analysis: Principal Component Analysis (PCA) and Centroid Factor Analysis (CFA) (Watts & Stenner, 2012), both of which are known to lead to similar results (Akhtar-Danesh, 2017a; Ramlo, 2016) and neither of which is deemed superior to the other (Akhtar-Danesh, 2017b). Therefore, data analysis began with an exploration of both approaches to understand the impact of analytical choice on the solution and to determine the number of factors to retain (Supplemental File). The Decision Criteria of Watts & Stenner (Galema et al., 2024; Watts & Stenner, 2012) (Table 1) was applied throughout this process to guide decision-making and ensure a robust final solution. Results from the CFA and PCA exploratory analyses offered similar explanatory power, easy of interpretability and comparable perspectives (Supplemental File). However, CFA allows for the application of abductive reasoning and selection of factors providing the most meaningful and plausible explanation (Ramlo, 2016). Therefore, CFA was selected for analysis.

**Table 1:** Watts & Stenner decision criteria for factor retention

**Post-Factor Extraction**	
Kaiser Guttman Criteria	Retain factors with an eigen value > 1, as those with an eigenvalue < 1 explain less variance than a single Q sort and are not deemed relevant for inclusion.
Verify cumulative variance is > 35%	This is considered evidence of a robust solution in Q-Methodology
Scree Test	Visually inspect the Catrell Scree Plot (PCA only) to determine where the slope of the eigen value line begins to flatten, as this indicated diminishing returns of explained variance.
**Post-Factor Rotation**	
Factor Loadings	Retain factors with three or more significant loadings (p < 0.001) as this represents stable underlying patterns rather than strong singular opinions.
Humphrey’s Rule	Retain factor where the cross-product of the two highest loadings on each factor exceeds twice the standard error (2×0.17 = 0.34), as this represents stable underlying patterns rather than strong singular opinions.

#### Centroid factor analysis

The correlation matrix was computed from the Q-sorts and CFA was applied to extract factors, eigen values (EV) and percentage of variance explained. In keeping with Watts & Stenner Decision Criteria (Watts & Stenner, 2012) (Table 1), Kaiser-Guttman Criteria was applied and all factors with EV > 1 were retained. This decision is based upon the fact that EVs < 1 represent less variance than a single Q-sort and are therefore not deemed relevant for inclusion (Watts & Stenner, 2012). Next, the cumulative variance of factors retained was verified to be greater than 35%, indicating a robust solution in Q-methodology (Watts & Stenner, 2012).

#### Varimax rotation

Factor rotation is the process of rotating the positioning of the Q-sorts, relative to one another, to enhance the factor structure (Ramlo, 2016). This allows the most meaningful view of the data to be achieved and improve intrepretability (Watts & Stenner, 2012). In Q-methodology, factor rotation occurs through one of two methods: varimax or theoretical rotation (Watts & Stenner, 2012). Varimax rotation is an orthogonal rotation which assumes factors are not correlated and enables the emergence of distinct patterns (Ramlo, 2016). Theoretical rotation is performed manually based upon prior theory or substantive knowledge (Watts & Stenner, 2012). As this was an exploratory study, substantive knowledge was limited. In addition, the aim of this investigation was to illuminate distinct patterns of thinking. Therefore, varimax rotation was chosen to optimize the loading of Q-sorts upon the factor that they demonstrated the highest correlation, while minimizing confounding and enhancing interpretability. In keeping with Watts & Stenner Decision Criteria (Watts & Stenner, 2012), factors were retained after rotation if they demonstrated three or more significant loadings (p < 0.001) and met Humphrey’s Rule - the cross-product of the two highest loadings on each factor exceeds twice the standard error (2×0.17 = 0.34). Each of these conditions help ensure that stable underlying patterns are represented in the data rather than strong singular opinions of individuals (Watts & Stenner, 2012).

### Interpreting the data

Factors were initially interpreted through factor arrays, crib sheets, and distinguishing and consensus statements (Supplemental File). Factor arrays are hypothetical Q-sorts that represent the composite of all Q-sorts which loaded significantly on a factor. Distinguishing and consensus statements are list of statements that set one perspective apart from another or bind all perspectives together. Crib sheets are lists of statements that distinguish one factor from another. These include the statements ranked highest and lowest within each factor, and statements that are ranked relatively higher or lower compared to other factors. Narrative descriptions representing the unique perspective of each factor were initially drafted based on this information and discussed among the research team when selecting the analytical approach, number of factors to retain and when identifying further points of interest for exploration through qualitative data.

#### Sensitivity analysis of factor loadings

The degree to which a participant Q-sort contributes to the explained variance of a factor is derived from its factor loading value, which represent how closely the Q-sorts correlates with a factor (Watts & Stenner, 2012). Factor loading values range from + 1 (perfect correlation) to − 1 (perfect opposition), with 0 indicating no correlation. As Varimax rotation assumes non-correlation between factors, Q-sorts can only be assigned to one factor. Therefore, they are assigned to the factor they are most correlated with, regardless of the level of correlation or the extent of variance explained. This means that some Q-sorts are much less strongly aligned with the factor that they load upon than others and a decision must be made regarding what factor loading value is close enough to be considered closely aligned with a factor. There is no gold standard for determination of factor loading cut-off values (Watts & Stenner, 2012). Therefore, a sensitivity analysis of factor-loading cut-off values (none, 0.40, 0.50 and 0.55) was conducted to observe the effect on solution clarity, interpretability and reliability of the factor estimates (Supplemental File). Following completion of the sensitivity analysis, narrative descriptions of the four perspectives were updated accordingly based on these results.

#### Qualitative data

 Qualitative data was gathered during post-Q-sort interviews. Interviews were audio recorded with QuickTime software (QuickTime player, 1992) and transcribed by a professional transcription service which states in its contract that data confidentially will be upheld. Transcriptions were inductively analyzed to highlight nuance and complexity in the study themes: awareness, motivation and engagement in cost-conscious decision-making. This data was used to differentiate meaning within and between distinct perspectives during the exploratory analysis, factor interpretation and following completion of the sensitivity analysis. Final narrative descriptions were approved by all members of the research team.

### Reporting results

The Q-methodology used in this study and resulting data are reported in accordance with the Checklist for reporting a Q-methodology study (Dieteren et al., 2023) and include participant characteristics, factor arrays, distinguishing and consensus statements, (Supplemental File) and narrative descriptions for each perspective. Narration of each perspective includes explained variance, characteristics of the educators composing the perspectives and descriptions of educator awareness, motivation and engagement in cost-conscious decision-making.

### Reflexivity

The research team is composed of consisted of members with different backgrounds, relevant to this study. JY is a former physical therapist and manager, who is currently a PhD candidate conducting exploratory research on the costs of HPE. BL is Assistant Professor of Methodology and Statistics who contributed expertise in mixed methodology. ME is Professor of Health Professions Education, former Scientific director of the Institute for Education and current Vice-Dean of Education at the FHML at University of Maastricht, with expertise in quality culture and management in higher education. SE is Professor of Health Technology Assessment and Scientific Director of Care and Public Health Research Institute, with expertise in health economic research. AP is Professor of Economics of Education and Healthcare, whose expertise lies at the intersection of HPE and economics. All authors share an interest in addressing resource-scarcity issues in HPE through cost-conscious decision-making. JY, ME, SE and AG have previously collaborated on an exploratory investigation of costs and cost identification methods in HPE (Yaros et al., 2023,2024), which informed the conceptual design and motivation for this study. All authors are also active educators and aware that experiences derived from their different roles and positions have shaped their own perspectives. However, the diversity in backgrounds helped to broadly reflect on the data of this study.

## Results

### Participant characteristics

Twenty-nine educators participated in this study. They held professional titles ranging from PhD Candidate to Full Professor, had 1–29 years of experience as educators and held a diversity of educational responsibilities. In general, educators’ self-perceived level of experience with educational budgeting and economics of education was limited (rated as none to novice) and varied with educational experience.

Four participants were excluded from analysis following a sensitivity analysis of factor-loading cut-off values, as their factor-loading score did not meet the threshold of 0.50, as described below in Factor Solution. Characteristics of the 25 participants who were included in analysis are described in Table 2 and summarized in narrative format.

**Table 2:** Characteristics of the 25 participants included in analysis of the perspectives

Perspectives	Unaware Doubters	Cautious Realists	Pragmatic Supporters	Empowered Agents	Totals
**Sample** (n (%))					
Total Participants	4 (14)	8 (28)	10 (34)	7 (24)	29 (100)
Excluded from Analysis	1 (3.5)	0 (0)	2 (7)	1 (3.5)	4 (14)
Included in Analysis	3 (12)	8 (32)	8 (32)	6 (24)	25 (100)
**Professional Title** (n (%))					
PhD Candidate	1 (13)	1 (12.5)	2 (25)	-	4 (16)
Assistant Professor	-	2 (25)	4 (50)	1 (16.3)	7 (28)
Associate Professor	-	2 (25)	-	3 (50)	5 (20)
Full Professor	-	2 (25)	-	1 (16.3)	3 (12)
Other	2 (67)	1 (12.5)	2 (25)	1 (16.3)	6 (24)
**Doman of Educational Responsibilities**^**1**^ (n (%))					
Health Science	1 (13)	5 (62.5)	5 (62.5)	3 (50)	13 (52)
Biomedical Sciences	2 (67)	3 (37.5)	5 (62.5)	2 (33.3)	12 (48)
Medicine	3 (100)	7 (87.5)	4 (50)	4 (66.6)	18 (72)
**Educational Experience** (median (spread))					
Years	4 (1–6)	25 (6–29)	6 (1–20)	16 (11–21)	12 (1–29)
**Level of Educational Experience** (n (%))					
Novice	3 (100)	1 (12.5)	4 (50)	-	8 (32)
Experienced	-	2 (25)	3 (38)	3 (50)	8 (32)
Expert	-	5 (62.5)	1 (12.5)	3 (50)	9 (36)
**Type of Educational Responsibilities**^**1, 2**^ (n (%))					
Delivery	3 (100)	8 (100)	8 (100)	6 (100)	25 (100)
Development & Organization	2 (67)	7 (88)	4 (50)	5 (83)	18 (72)
Oversight	2 (67)	5 (63)	2 (25)	6 (100)	15 (60)
**Number of Educational Responsiblities**^**2**^					
Single	1 (12)	1 (12.5)	3 (38)	-	5 (20)
Dual	-	2 (25)	4 (50)	1 (16)	7 (28)
Triple	2 (67)	5 (62.5)	1 (12.5)	5 (84)	13 (52)
**Time Devoted to Education**^**3**^ (median (spread))					
Percentage of Work Time	30 (10–100)	47.5 (10–100)	17.5 (0–100)	60 (30–90)	50 (0–100)
**Self-Perceived Experience with Educational Budgeting** (n (%))					
None	2 (67)	2 (25)	4 (50)	-	8 (32)
Novice	1 (13)	2 (25)	2 (25)	1 (16.6)	6 (24)
Experienced	-	3 (37.5)	2 (25)	5 (83.3)	10 (40)
Expert	-	1 (12.5)	-	-	1 (4)
**Self-Perceived Experience with Economic Concepts of Education** (n (%))					
None	1 (13)	3(37.5)	4 (50)	-	8 (32)
Novice	2 (67)	4 (50)	4 (50)	5 (83.3)	15 (60)
Experienced	-	1 (12.5)	-	1 (16.6)	2 (8)
Expert	-	-	-	-	-

1. Totals exceed 100% as participants may hold educational responsibilities in multiple categories

2. Based on past three years

3. Based on contractual hours

### Factor solution

Brown Centroid Factor Analysis with Varimax rotation and a factor-loading cut-off value of 0.50 resulted in a 6-factor solution with three consensus statements. Two additional patterns of consensus emerged from the qualitative data. Two factors were discarded for failure to meet the Watts & Stenner Decision Criteria (Watts & Stenner, 2012) (Kaiser Guttman Criteria, three or more significant loadings on a factor, and Humphrey’s Rule), resulting in a final solution of four distinct factors. Four participant Q-sorts failed to meet the factor loading cut-off value of 0.50 and were excluded from analysis and interpretation of the four distinct factors. Their exclusion did not alter the final solution of four distinct factors but did improve the clarity and ease of interpretation the four-factor solution. The final solution was derived from 25 participant Q-sorts which loaded significantly on four factors and explained 62% of cumulative variance. Full factor arrays, correlations between factors, crib sheets and distinguishing and consensus statements can be found in the Supplemental File. The four final factors combined with data from the interviews formed the basis for the following four divergent educator perspectives on the use of costs and cost-conscious decision-making and their labeling.

## Divergent educator perspectives

### Characteristics of the four perspectives

Q-methodology aims to identify shared patterns of thinking rather than correlate or predict perspectives based on participant characteristics. However, understanding how individuals align with each perspective can aid in interpretation of the results. For this reason, we summarize the characteristics of the participants across the four perspectives (Table 2), while also cautioning against over-interpreting these associations.

The 25 participants included in the final four perspective solution varied in professional titles, years of educational experience and responsibilities, and self-perceived familiarity with budgeting and economic concepts. All participants were involved in educational delivery, however the majority held two or more educational responsibilities. Only novice educators working as PhD candidates and a single post-doctorate held singular responsibilities. *Unaware Doubters* were composed entirely of novices. *Cautious Realists* had the highest number of expert educators and the widest spread of years of educational experience. *Pragmatic Supporters* included no Associate or Full Professors but did represent all career stages and all three levels of involvement in educational responsibilities. *Empowered Agents* were equally split among experienced and expert educators and reported the most extensive involvement with educational responsibilities and the greatest familiarity with budgeting and economic concepts. Again, these descriptive patterns are not intended to imply predictive relationships, only to offer contextual insight into the four divergent perspectives.

### Unaware Doubters

This perspective explains 12% of study variance (EV = 3.63) and is composed of three novice educators with a median of four (spread of 1–6 years) years of educational experience. The highest levels of educational responsibilities within this group included educational oversight (n = 2, 66%) and educational delivery (n = 1, 33%). These educators contributed between 10–100% of their contracted work time towards education.

As their label implies, Unaware Doubters do not emphasize awareness of resource scarcity problems in HPE (such as personnel shortages) and strongly indicate a lack of exposure to demands for educational cost accountability, transparency and efficiency. They also strongly emphasize their lack of involvement in educational budgetary considerations. Furthermore, they do not currently consider how to optimize educational resource use such as personnel, materials, equipment or facilities, which one participant attributed to a lack of knowledge, “*I have no idea of the reality of things*” (P15).

Unaware Doubters express moral opposition to the inclusion of cost information in educational decisions as they feel that costs are reductionist and can only tell us the price of things, not the value of education - which one participant sees as societal impact, and cautions *“I think we already have quite some difficulty in making that insightful, what the societal impact is”* (P16), let alone linking that to educational costs. Additionally, they feel more doubtful than any other perspective that it would be possible to capture the nuance and complexity of education in terms of costs alone, for *“you can never measure it in a way that is going to be comprehensive of what is actually learned or what the person that was enrolled or that took part in that little educational piece learned or experienced” (P15).* In this regard, they represent the perspective with the least motivation for cost-conscious decision-making.

Lack of motivation to participate in cost-conscious decision-making may arise from the fact that none of these educators felt they had the power to influence educational costs. Despite uniform lack of agency, the desire for influence and responsibility over cost considerations was differently experienced, with one novice educator questioning “*what would be the use of me getting involved in that because I don’t see myself in this position being able to change anything whatsoever*” (P20), while others felt they either did not have adequate experience or did not want additional tasks to be added to an already overburden agenda– “*No, it’s not something I also want as an additional task”* (P16) In addition to being disinclined to engage in cost-conscious decision-making, Unaware Doubters were somewhat distrustful of educational administrators’ intentions to adopting such practices. On a more positive note, they did feel that other more experienced educational designers should be included in educational cost conversations with financial experts and educational administrators and that they would need guidance if they were required to participate themselves.

### Cautious realists

This perspective explains 16% of study variance (EV = 9.24) and includes eight diverse educators. The majority (n = 5, 62.5%) are expert level educators. They have a median of 25 (spread 6–29) years of experience. The highest level of educational responsibilities for this group is educational oversight (n = 5, 63%), followed by development and organization (n = 7, 88%), then delivery (n = 8, 100%). The percentage of their work time contracted for education ranged widely from 10–100%.

Despite being relatively unexposed to cost considerations, this group of educators did express awareness of resource scarcity explaining they personally find themselves impacted by financial and staffing constraints. “*They impacted me very practically, in me not being able to find sufficient teachers for the courses that I ran … people not being able to pick up things for each other … .it remains silent for a longer period of time. It happens that I get help eventually, but it takes longer than you would hope in an organization that has so many people in it. Yes, and then I think we also see it in people getting sick, getting burnt out, or whatever it is they experience” (P29).*

Cautious Realists strongly emphasize that their role as educators is first and foremost an obligation to providing high-quality education for students and they feel that education should offer high value to society. They also recognize that almost all choices have financial consequences and that financial limits must be respected to maintain and sustain systems of education. By and large, they expressed a high level of willingness to consider cost information, if clear and transparent policies existed to detail how, when and why cost information would be applied in decision-making. Yet, as one expert educator cautioned “*I would be willing to consider, but that is not the same thing as knowing how*” (P3). Despite willingness, they emphasized more distrust than any other perspective of educational administrators’ intentions to adopt cost-conscious decision-making. Other obstacles included not having any influence on budgeting (of educational hours) as it is “*already 95% given*” (P12) or lack of exposure to budgeting information and therefore a tendency to more strongly align with students’ educational needs than educational costs or financing. “*I teach the amount of hours that I feel I need to help students the best way possible. Hence, I think more about the perspective from the student than the perspective of our university*” (P23).

This group offered several strategies to enable their participation in cost considerations. Many spoke of a desire for “*more transparency, more openness*” (P19) and “*rationale for why things are happening*” (P29) from upper management, while others voiced the importance of establishing guiding principles, emphasizing that “*we cannot talk about costs if we haven’t spoken about our morals, about what academic education is*” (P19). Lastly, whether they are personally involved in cost-conscious decision-making or not, they also felt this was a multi-disciplinary endeavor that must include educational designers and administrators, along with financial experts.

### Pragmatic supporters

This perspective explains 16% of study variance (EV = 1.71) and includes eight diverse educators. Novice educators composed half of this group (n = 4, 50%), yet as a group they have a median of six and a spread of 1–20 years of educational experience. Their highest educational responsibilities were evenly distributed across delivery (n = 6, 100%) development and organization (n = 5, 83%) and oversight (n = 6, 100%). Collectively, the percentage of their time contracted for education ranged widely from 0–100%.

These educators did not directly emphasize awareness of resource scarcity in higher education, choosing instead to give greater importance to their obligation as educators to provide high-quality education for students. Even so, in conversation with Pragmatic Supporters awareness of resource scarcity emerged implicitly through repeated mention of the mismatch between educational workload and available work hours - highlighting the steadily rising pressure in this profession. “*We put in a lot of extra time, me and many others … .all the work that you have to do yourself goes into the evening hours*” (P7).

Similar with Unaware Doubters, Pragmatic Supporters felt that cost information alone would be unable to capture the nuance and complexity of educational value, which they saw “*an investment in society as a whole*” (P26). Yet, they were the one perspective that emphasized the potential for improving educational value through the comparison of educational costs and educational quality. They also expressed their support of cost-conscious decision-making by positively agreeing to consider cost information in summary reports and if there were clear and transparent policies detailing how, when and why it should be applied in decision-making. Accordingly, they were the only perspective to express trust of educational administrators’ intentions.

Pragmatic Supporters also mention several hinderances limiting their ability to engage with and act upon cost information. First, cost-conscious decision-making is not a skill or practice that is formally embedded in the educational training or the career development pathway, but rather a process learned implicitly along the way. “*This is something that I would love to have had six years ago when I started to just get the sense of, okay, what’s reasonable, what’s not reasonable, and perhaps how to navigate this. At least some statement of principles would be helpful*” (P11). Second, the cost information they do have access to is typically prepared to meet legal or financial criteria and has not been translated into the language of educators. “*It is commonly very jargony, very lengthy and dedicated, and not meant to be read by people who want a nice read. I think that is not so useful*” (P7). To individually overcome these challenges, Pragmatic Supporters seek clear and formalized guidelines and policies, and mentorship from more experienced individuals. While on a more global level, they are adamant that cost-conscious decision-making should be steered collaboratively by educational designers, administrators and financial experts.

### Empowered agents

This perspective explains 18% of study variance (EV = 1.56) and consists of six educators who are equally split between experienced (n = 3, 50%) and expert level educators (n = 3, 50%). They have a median of 16 (spread of 11–21) years of educational experience. The highest level of educational responsibilities held by all Empowered Agents (n = 6, 100%) is educational oversight. They contribute a median of 60 percent of their working time on education (spread 30–90%).

These educators already feel the demand for cost accountability and transparency and strongly emphasized their awareness of financial strain, with one educator commenting that “*the scarcity of resource hours, people and rooms*” (P1) is notable on a daily basis, while another stated that “*costs were constantly a problem but somehow we managed*” (P8) during program revisions. In addition, they felt more strongly than any other perspective that they already optimize educational resource use and have a feeling that they can influence educational costs.

Empowered Agents agree that educational costs must be considered. Motivation for these considerations ranged from an ambition to grow into higher educational roles, a desire to reduce work pressure, a responsibility to use public funds efficiently and to reduce educational inequities. Despite these inclinations, Empowered Agents ranked their willingness to consider educational costs via summary reports or in relation to clear policies lower than any other group. Furthermore, none have received any formal training in educational cost considerations, and all have had to develop the own systems and processes to navigate educational cost considerations. “*There are no guidelines … I was a bit surprised that we have to figure it out ourselves*” (P13). In addition, to a lack of desire for additional oversight regarding how to go about their decision-making, Empowered Agents also express mild distrust of educational administrators’ intentions for adopting cost-conscious decision-making.

To enhance their engagement, Empowered Agents seek insight into the vision and strategy that guides educational decision-making in the upper echelons. As one expert educator indicated, there may be “*transparency about the expectations, but it’s not necessarily a transparency about what people are actually doing or why these decisions are being made or what the rationale is for that sort of thing*” (P17). Taking this idea one step further, another expert educator indicated a need to clarify role responsibility, stating “*I would like to see conversations about what is the role, the vision, and who does what*” (P28). In addition to building a shared vision of cost-conscious decision-making, Empowered Agents also called for training workshops earlier in their career pathway, guidelines which are formalized, but not prescriptive, and the development of a shared and meaningful language so that educational designers, administrators and financial experts can work together to address resource scarcity issues in HPE.

## Discussion

This investigation utilized Q Methodology and the Integrated Change Model to map educator perspectives on costs and cost-conscious decision-making in HPE. We included novice, experienced and expert educators whose educational responsibilities included delivery, development and organization and oversight of education. Our study identified four distinct educator perspectives, labeled as: Unaware Doubters, Cautious Realists, Pragmatic Supporters and Empowered Agents. These perspectives demonstrate variation in cost awareness, motivation and engagement among HPE educators and indicate a need for perspective-driven approaches to foster system-wide, cost-consciousness in HPE decision-making.

As this was an exploratory investigation of an emerging topic, there is only limited evidence with which to compare our findings (Cleland et al., 2022). To the best of our knowledge no other investigation has explored cost-consciousness among HPE educators, whereas this topic does appear in literature pertaining to healthcare providers (Carroll et al., 2024; Cooke, 2010; Leep Hunderfund et al., 2017; Moleman et al., 2021,2022; Mordang et al., 2020; Stammen et al., 2019; Stammen et al., 2015). While a closely related field, those investigations focused upon the costs of healthcare delivery decisions, not the costs associated with HPE decisions, and were deemed irrelevant for comparison. Henceforth, we will focus on potential reasons and implications for the differences in awareness, motivation and engagement between the four educator perspectives and, where possible, make comparisons with existing evidence.

### Reasons and implications

Awareness of resource scarcity varied in origin and extent of awareness. Unaware Doubters lacked awareness of resource scarcity, which is likely embedded in the fact that they held the fewest years of educational experience (median of 4 years) and had the least percentage of their time contracted for educational responsibilities. It may be their status as early career educators intentionally shields them from exposure to extraneous tasks such as cost considerations to allow them time to develop core educator competencies instead. Given the importance of protecting this developmental phase, it is important that educational institutes seeking to foster cost-conscious decision-making explore who exactly needs to know what, when knowledge acquisition should begin, and how much needs to be known at each stage of development. Answers to these questions can help institutions develop a progressive roadmap towards cost-conscious decision-making.

Pragmatic Supporters, with a median of 6 years of educational experience also did not emphasize awareness of resource scarcity, yet they did strongly emphasize their experience of steadily increasing work pressure. That they fail to recognize increased work pressure as a results of resource scarcity indicates that the link between resource scarcity issues and increasing work pressure has not yet been made explicit. Cautious Realists and Empowered Agents, the two groups with the most experience in education (median of 25 and 16 years, respectively) did demonstrate awareness of resource scarcity, albeit in slightly different ways. For Cautious Realists, awareness was largely limited to the challenges they faced when attempting to secure adequate personnel to fulfil educational roles in their courses and programs, whereas Empowered Agents also indicated this and expressed additional awareness of financial limitations. For Cautious Realists, Pragmatic Supporters and Empowered Agents, awareness may be best fostered by directly engaging them in conversations and explicitly linking educational costs with resource scarcity issues as well as their potential impacts on educational decisions.

Regarding motivation to participate in cost considerations, we found several similarities and differences between the educators who participated in this study and the educational thought leaders explored by Cleland et. al (2022). First, educators in both studies recognized the importance of managing educational costs, while stressing their primary allegiance was to education and ensuring educational quality. Second, all felt cost information was reductionist and unlikely to capture the true value or worth of HPE. This may partly be due to their tendency to view the value of HPE in terms of higher order goals, such as the development of human potential and advancement of society, which indeed would be difficult to capture in terms of costs alone, if at all. However, these sentiments also mark an opportunity to increase HPE educators’ awareness of the purpose and potential of integrating educational costs into HPE decision-making, including the use of full economic evaluations which integrate both educational costs and effects into analyses.

While thought leaders expressed a fear of what could happen to their autonomy if asked to engage in cost-conscious decision-making (Cleland et al., 2022), most educators in our study took a slightly different view and underscored what was necessary to preserve their autonomy: cost conversations should be open, transparent, and balanced rather than prescriptive. In this way, educators in our study recognized the potential for cost considerations to help them align their decisions with system-level priorities and the possibility of positively impacting education. The source of this difference likely arises from differences in our line of questioning, as we specifically asked educators what it would take for them to engage in cost-conscious decision-making. The implication for educational institutes is that it is important to frame discussions of, or introductions to, cost-conscious decision-making in a collaborative and forward-thinking manner. Lastly, we found conflicting evidence for the suggestion that educator motivation for cost conscious decision-making may be hampered by distrust of those requesting cost-conscious decision-making (Walsh, 2013). In our investigation, this only held true for the least inclined (Unaware Doubters and Cautious Realists), who felt they had the least influence over upper management decisions pertaining to costs. By contrast, Pragmatic Supporters, who had the most positive outlook of all perspectives, expressed trust in their administrators and Empowered Agents, who were the most actively engaged in cost-conscious decision-making, reserved mild distrust. This indicates that individual outlook and proximity to, or involvement in, educational cost considerations with upper management each impact trust in administrators. Consequently, this implies that there is potential to build trust and momentum for educator involvement through increased interaction with upper management.

Regarding engagement, our findings were in keeping with Cleland et. al., (2022) that no educators had received formal training to guide educational cost considerations and were either were self-taught or learned via mentorship of more experienced colleagues. As Walsh suggests, this is likely due to the lack of cost-conscious training along professional development pathways in education (Walsh, 2013). As indicated in our study and the Cleland study (Cleland et al., 2022), the uptake of this responsibility appears to occur primarily out of necessity as educators advance into managerial and administrative roles. Thus, educator engagement in cost-conscious decision-making is primarily driven by extrinsic rather than intrinsic motivation. These findings confirm a need to develop and integrate such trainings into continuing professional development and foundational training programs and the need to incentivize participation.

A variety of needs emerged to enable participation in cost-conscious decision-making among educators. Unaware Doubters are not interested in participating, which is again likely due to their status as novice educators. Cautious Realists desired greater transparency, some degree of influence, and the establishment of principals to guide the application of cost-conscious decision-making. Pragmatic Supporters felt they had been ill-prepared to take cost-conscious decisions and expressed a strong desire for guidance, training and mentorship. Empowered Agents, who had already established their own practices of making cost-conscious decisions, placed greater emphasis on transparent communication from leadership, clarification of roles and responsibilities and the establishment of an institutional vision on cost considerations. Lastly, all educators felt that educational designers must be included in the cost-conscious decision-making processes alongside educational administrators and financial experts. This indicates that these educators are interested in having a seat at the table for this discussion. However, they also cautioned that for this to be an effective collaboration, each stakeholder must have basic knowledge of the others’ discipline, and a common language must be developed to foster information exchange.

### Strengths and limitations

Although the costs of HPE are a concern for many stakeholders, evidence on educator perspectives regarding the use of cost information in educational decision-making remains sparse. Previous research in this area is limited to a single, qualitative study examining educational leaders’ attitudes toward cost and value research. Our investigation addresses this gap by being the first to explore educators’ awareness, motivation and engagement towards cost-conscious decision-making in HPE, thereby expanding the scope of understanding of this critical topic. Additionally, our study enhances the robustness of the evidence base by including a larger number and more diverse sample of educators and by utilizing mixed methods to triangulate our findings. Finally, we have introduced a model of educator perspectives that can be tested in different settings and contexts and used by educational leaders to foster system-wide cost-conscious decision-making among educators holding different levels of awareness, motivation and engagement.

The findings of this study should be interpreted with the following limitations in mind. First, development of the statement set is driven by researcher judgment, which may have influenced its balance and comprehensiveness or introduced subjectivity into the data. To mitigate these potentialities, the statement set underwent pilot testing and subsequent adaptation, while participants who completed the Q-sort were asked whether any ideas or sentiments were missing from the statements. Most participants reported the statement set was comprehensive and complete. Participants who suggested novel viewpoints, typically merged two existing statements or reworded statements to add specificity. Based on this feedback, the statement set was deemed broadly representative of the current educator discourse on this topic. Second, the use of a forced-choice distribution to rank statements required participant to prioritize their responses, mimicking real-world decision-making constraints. However, this approach may have introduced bias by compelling participants to rank statements in a way that may not fully align with their perspectives. To address this, participants were encouraged to continue adjusting and rearranging the statements until the rankings best represented their unique viewpoints. As the aim of this study was to identify patterns of consensus, the potential impact of this limitation is expected to be minimal. Third, this study was conducted at a single educational institute in the Netherlands, which employs small-scale, problem-based learning. The budgeting experience of educators in this context is largely concentrated upon personnel time through the allocation of education hours. As such, the findings may not be generalizable to other educational institutions with different educational systems or resource allocation practices. Lastly, Q-methodology is a form of by-person factor analysis which aims to identify distinct patterns of thinking among a set of participants, not to predict participant perspectives based on distinct characteristics. Future research is needed to explore whether and how characteristics such as professional role, educational responsibilities and years of experience are associated with the four perspectives identified in this study.

### Conclusions

This investigation provides an overview of varying HPE educator perspectives on costs and cost-conscious decision-making in education. Such information can be used by educational institutes to develop tailored strategies aimed towards improving awareness, incentivizing motivation, building capacity for cost-consciousness and enabling widespread participation in cost-consciousness decision-making among HPE educators.

## Electronic supplementary material

Below is the link to the electronic supplementary material.

Supplementary Material 1

## Data Availability

Data is available upon request made in writing via email to the primary author.
