# Supplementary material for: Educator perspectives on costs and cost-conscious decision-making in health professions education: a Q-Method study
Source: Adv Health Sci Educ Theory Pract. 2025 Aug 6;31(2):619–40. doi: 10.1007/s10459-025-10463-2 (PMC13046565; doi:10.1007/s10459-025-10463-2)
Supplement: Supplementary file 1 — Supplementary Material 1 [file 10459_2025_10463_MOESM1_ESM.docx]

**Article Title**: Educator Perspectives on Costs and Cost-Conscious Decision-Making in Health Professions Education: A Q-Method Study

**Journal:** Advances in Health Sciences Education

**Authorship**: Jennifer Yaros^1^, Mirjam oude Egbrink^2^, Benedikt Langenberg^3^, Silvia Evers^4^, Aggie Paulus^5^

1. J.K. Yaros, Maastricht University, Faculty of Health Medicine and Life Sciences, School of Health Professions Education (SHE), Department of Health Services Research (HSR), Maastricht, The Netherlands. https://orcid.org/0000-0002-4976-5202

2. M.G.A. oude Egbrink, Maastricht University, Faculty of Health Medicine and Life Sciences, School of Health Professions Education (SHE), Department of Physiology, Maastricht, The Netherlands. https://orcid.org/0000-0002-5530-6598

3. B. Langenberg, Maastricht University, Faculty of Health Medicine and Life Sciences, Care and Public Health Research Institute (CAPHRI), Department of Methodology and Statistics, Maastricht, The Netherlands. https://orcid.org/0000-0002-4757-0698

4. S.M.A.A. Evers, Maastricht University, Faculty of Health Medicine and Life Sciences, Care and Public Health Research Institute (CAPHRI), Department of Health Services Research, Maastricht, The Netherlands; Trimbos Institute, Center for Economic Evaluation and Machine Learning, Netherlands Institute of Mental Health and Addiction, Utrecht, The Netherlands. https://orcid.org/0000-0003-1026-570X

5. A.T.G. Paulus, Maastricht University, Faculty of Health Medicine and Life Sciences, School of Health Professions Education (SHE); and Care and Public Health Research Institute (CAPHRI), Department of Health Services Research, Maastricht, The Netherlands. <https://orcid.org/0000-0002-2086-6106>

**Corresponding Author**: Jennifer Yaros, [j.yaros@maastrichtuniversity.nl](mailto:j.yaros@maastrichtuniversity.nl)

**Supplementary file 1. Statement Set**

1. I find economic concepts difficult, complex and confusing.
2. I already try to optimize educational costs such as time, personnel, materials, equipment and facilities as efficiently and effectively as possible.
3. I would need guidelines outlining what is expected from me if I had to consider the educational costs associated with my roles.
4. I’m worried that educational administrators will place too much emphasis on educational costs and not enough emphasis on educational outcomes or institutional objectives.
5. I frequently think about how I can limit educational costs.
6. I currently use budgetary, cost or financial information to coordinate, manage and execute educational responsibilities.
7. I find it really uncomfortable to talk about educational costs.
8. I think cost information could be useful when making choices related to high-cost, high-volume and high-priority educational objectives.
9. I think we have an obligation to the students to provide high-quality education at a reasonable cost.
10. I think costs are a moralistically flawed way to evaluate educational choices.
11. I think would be impossible capture the nuance and complexity of education in terms of costs alone.
12. I think the costs of education should only be considered by economic, accounting, or financial experts and educational administrators.
13. I am aware that institutes of higher education are experiencing budget shortfalls.
14. I think by comparing cost information with educational outcomes we can improve educational quality.
15. I feel that conversations about costs always precede budget cuts.
16. I already feel the demand for cost accountability, transparency and efficiency in education.
17. I think that by considering cost information we can free up under-utilized resources for other uses.
18. I think the benefit of education to society is immeasurable and worth whatever it costs.
19. I think that cost information will only tell us the price of something, not its value or worth.
20. I feel uncertain by what is meant by “educational costs.”
21. I would be willing to consider costs if there were clear and transparent policies detailing how, when, and why cost information would be applied in decision-making.
22. I see cost information as an opportunity for me to negotiate with others in a persuasive manner.
23. I’m afraid cost information will be used against us. If we manage to save money one year, we’ll end up receiving less money the next year.
24. I think cost information can be useful in securing educational funding.
25. I think managing educational costs is a multi-disciplinary effort.
26. I would consider educational costs if the information was presented to me in summary reports.
27. I’m worried that educational innovation will suffer if we start talking about costs.
28. I think cost information is a bit of a necessary evil.
29. I already see financial strain beginning to impact educational activities around me.
30. I don’t care about the costs of education.
31. I am in a position with the means to influence educational costs.
32. I think almost every choice has some financial consequences.
33. I think everyone supports the idea of reducing educational costs.
34. I am aware that institutes of higher education are struggling with personnel shortages.

**Supplementary file 2. Interview Questions**

**Specific**

- Can you explain to me why you’ve placed these statements at the extremes (+4 / -4) ?
- Are there any other statements that you feel strongly about? Can you tell me why you feel strongly about these statements?
- Optional: Can you tell me about why you placed these statements here and here? (indicate unusual or unexpectedly placed statements).
- Are there any other statements you would like to comment on?
- Were there any statements that you did not quite understand?
- Was there anything you felt was missing from these statements?
- If you could create one additional statement, what would it be?

**General**

- Would you share with me your understanding of using cost information when making educational choices? This may include
  - the types of costs
  - the cost consequences
  - who you feel should be involved in educational cost considerations
- Can you tell me how ready you feel to incorporate cost information into educational choices? Or what kind of support you feel is necessary to do so?

**Open**

- Any additional questions that come up in the context of the interview
- Is there anything else you wish to share?

**Supplementary file 3. Exploratory Analysis**

The method of analysis and number of factors to accept in the solution was determined though an exploratory analysis comparing the output of principal component analysis (PCA) and centroid factor analysis (CFA) with 2 – 8 factors retained in the solution. The decision-criteria of Watts & Stenner^1,2^ were applied throughout this process to inform the decision.

**Watts & Stenner Decision Criteria**

1. **Conduct Inverted Factor Analysis with PCA or CFA**

- Apply the Kaiser-Guttman Criteria and retain all factors with Eigenvalues (EV) >1. This decision is based upon the fact that EVs < 1 represent less variance in the data than a single q-sort and are therefore not deemed statistically significant for inclusion.
- Verify that retained factors explain a cumulative variance higher than 35%, which is considered a robust solution in Q-methodology.
- Examine the Cattell Scree Plot (PCA only) to visually determine where the slope of EVs begins to flatten, which is indicative of diminishing returns of explained variance.

Watts SS, Paul. *Doing Q Methodological Research. Theory, Method and Intrepretation*. Sage; 2012.

^2^ Galema G, Schönrock-Adema J, Jaarsma D, Wietasch G. Patterns of Medical Residents' Preferences for Organizational Socialization Strategies to Facilitate Their Transitions: A Q-study. *Perspect Med Educ*. 2024;13(1):169-181. doi:10.5334/pme.1189

1. **Conduct Varimax Factor Rotation**

- Retain factors that demonstrated three or more significant loadings (p<0.001), as this represents stable underlying patterns rather than strong singular opinions.
- Apply Humphrey’s Rule to verify that the cross-product of the two highest loadings for each factor exceed twice the standard error (2x0.17=0.34). This indicates clusters of shared opinion (factors) which should be retained and eliminates individual opinions that should not be retained.

1. **Examine Factor Arrays**

- Factor arrays are hypothetical q-sorts that represent a composite of all q-sorts loading on a factor. For factors to be accepted into the solution, the factor arrays should add substantive explanatory value while allowing for ease of interpretability.

1. **Decision**
   - Based on the above criteria, the Brown CFA 6-Factor and PCA 3-Factor solutions both meet the decision-criteria, offer substantive explanatory power, ease of interpretability and result in comparable typologies. Given the uniformity of these results, the Brown CFA 4-Factor model with Varimax rotation was chosen as it provided greater nuance and complexity in factor interpretation.

**Decision Criteria**

|  | **Brown CFA** | **PCA** |
| --- | --- | --- |
| Kaiser-Guttman Criteria (EV> 1) | 4 | 8 |
| Cumulative Variance Explained (%) | 52 | 56 |
| Cattell Scree Plot Test (PCA only) | N/A | 3 |
| Significant Loading at (p<0.001) for at least 3 q-sorts | 4 | 3 |
| Humphrey’s Rule: cross product of two highest loadings exceeds twice the standard error (SE = 0.17)) | 4 | 3 |
| Substantive Value & Interpretability | 4 | 3 |

**PCA with Varimax Rotation**

|  | 2 Factors | 3 Factors | 4 Factors | 5 Factors | 6 Factors | 8 Factors |
| --- | --- | --- | --- | --- | --- | --- |
| Q-sorts loading on a factor | 29 | 29 | 29 | 29 | 29 | 29 |
| Variance Explained (%) | 48 | 56 | 63 | 68 | 73 | 81 |
| Variance Explained (%) per Factor (F1-F2-F3-F4-F5-F6) | 34-14 | 34-14-8 | 34-14-8-7 | 34-14-8-7-5 | 34-14-8 -7-5-5 | 34-14-8-7-5-5-4-4 |
| Q-sorts per factor (F1-F2-F3- F4-F5-F6)) | 17-12 | 13-8-8 | 6-8-6-9 | 3-10-8-9-2 | 3-7-4-9-3-3 | 8-5-3-2-3-2-3-3 |
| Factors Retained in Model | 2 | 3 | 4 | 4 | 6 | 6 |
| Interpretation | Very simple, bi-polar factors | Clear and balanced, but only majority opinions represented | Explains more variance, offers more nuance, not all factors easy to interpret | No additional variance explained, less distinction between factors, increased difficulty interpreting | Explains additional variance, offers dominant and minority opinions, but remains difficult to interpret | Explains more variance but some factors have only one distinguishing statement. Results similar with previous models. |

**Brown CFA with Varimax Rotation**

|  | 2 Factors | 3 Factors | 4 Factors | 5 Factors | 6 Factors |
| --- | --- | --- | --- | --- | --- |
| Q-sorts loading on a factor | 29 | 29 | 29 | 29 | 29 |
| Variance Explained (%) | 45 | 45 | 52 | 52 | 57 |
| Variance Explained (%) per Factor (F1-F2-F3-F4-F5) | 32-3 | 32-13-0 | 32-13-1-6 | 32-13-1-6-0 | 32-13-1-6-0-5 |
| Q-sorts per factor (F1-F2-F3-F4-F5-F6) | 16-13-12 | 16-13-11 | 11- 7-0-11 | 11-7-0 -11-0 | 8-4-0-10-0-7 |
| Factors Retained in Model | 2 | 2 | 3 | 3 | 4 |
| Interpretation | Very simple, bi-polar model | No new factors added to the model | Explains more variance, offers slightly more nuance | No new factors added to model | Explains more variance, similar results as PCA 3F, but offers greater nuance and ease of interpretation |

**Supplementary file 4. Factor Arrays**

**
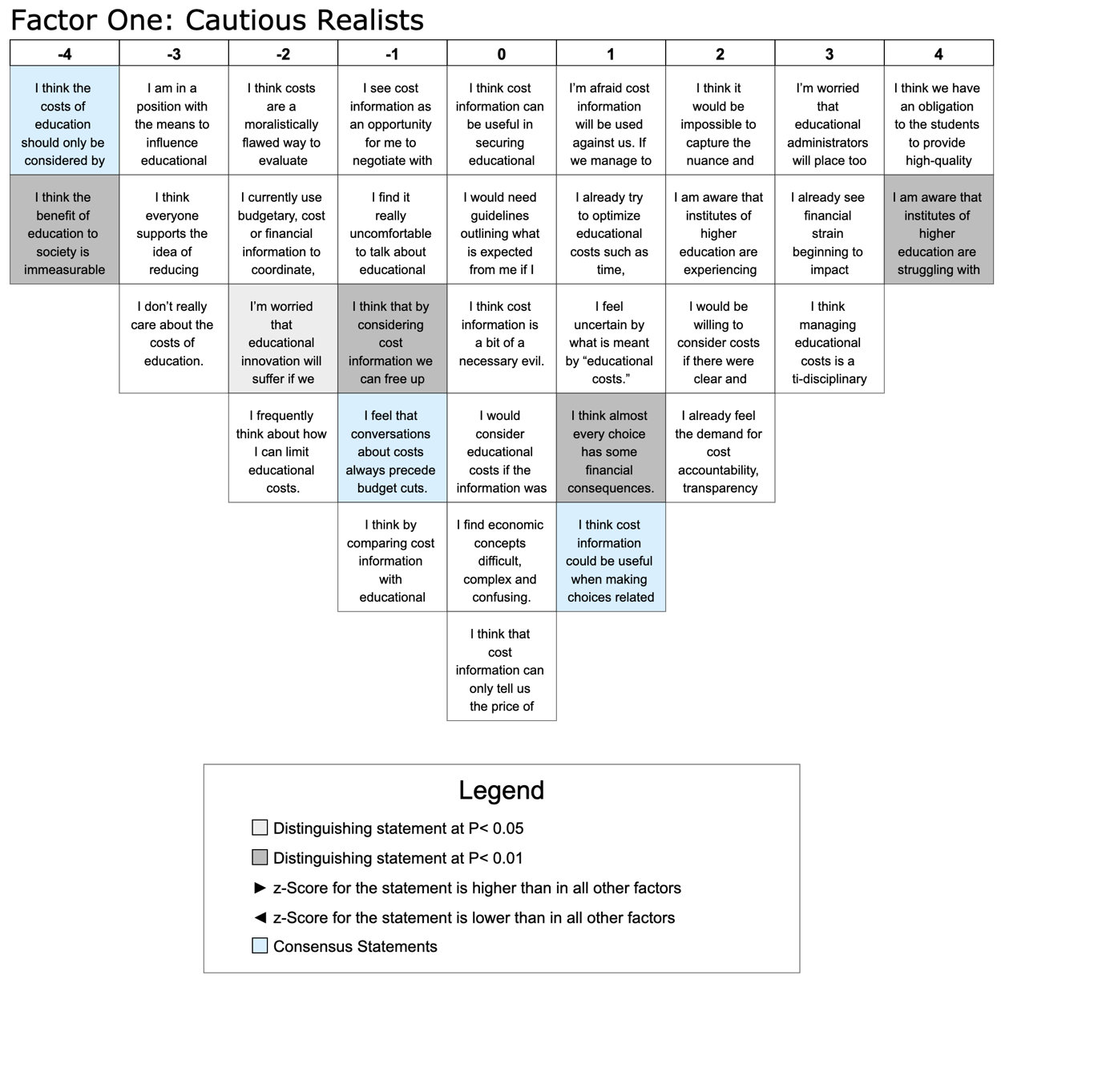

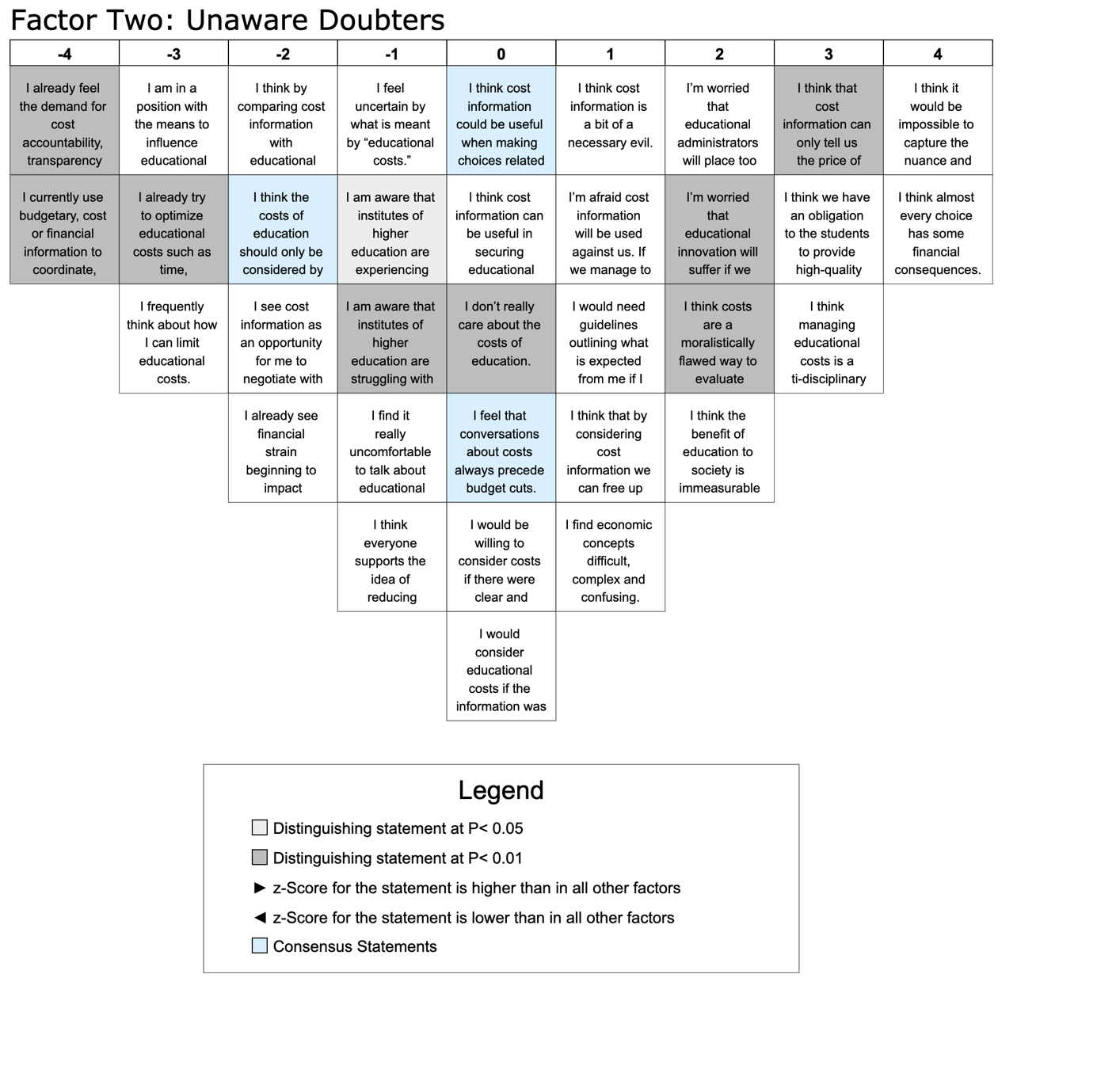

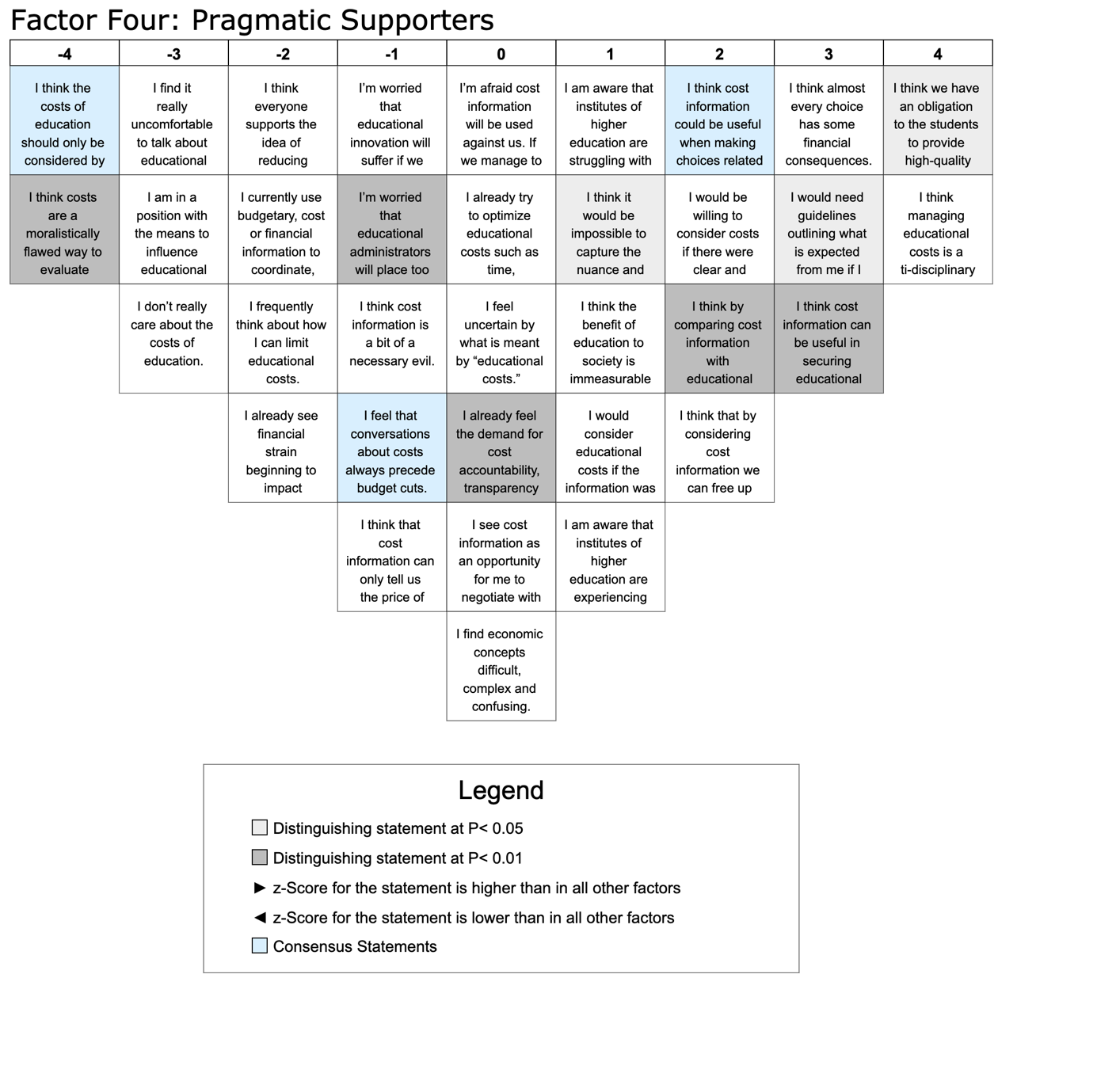
**

**
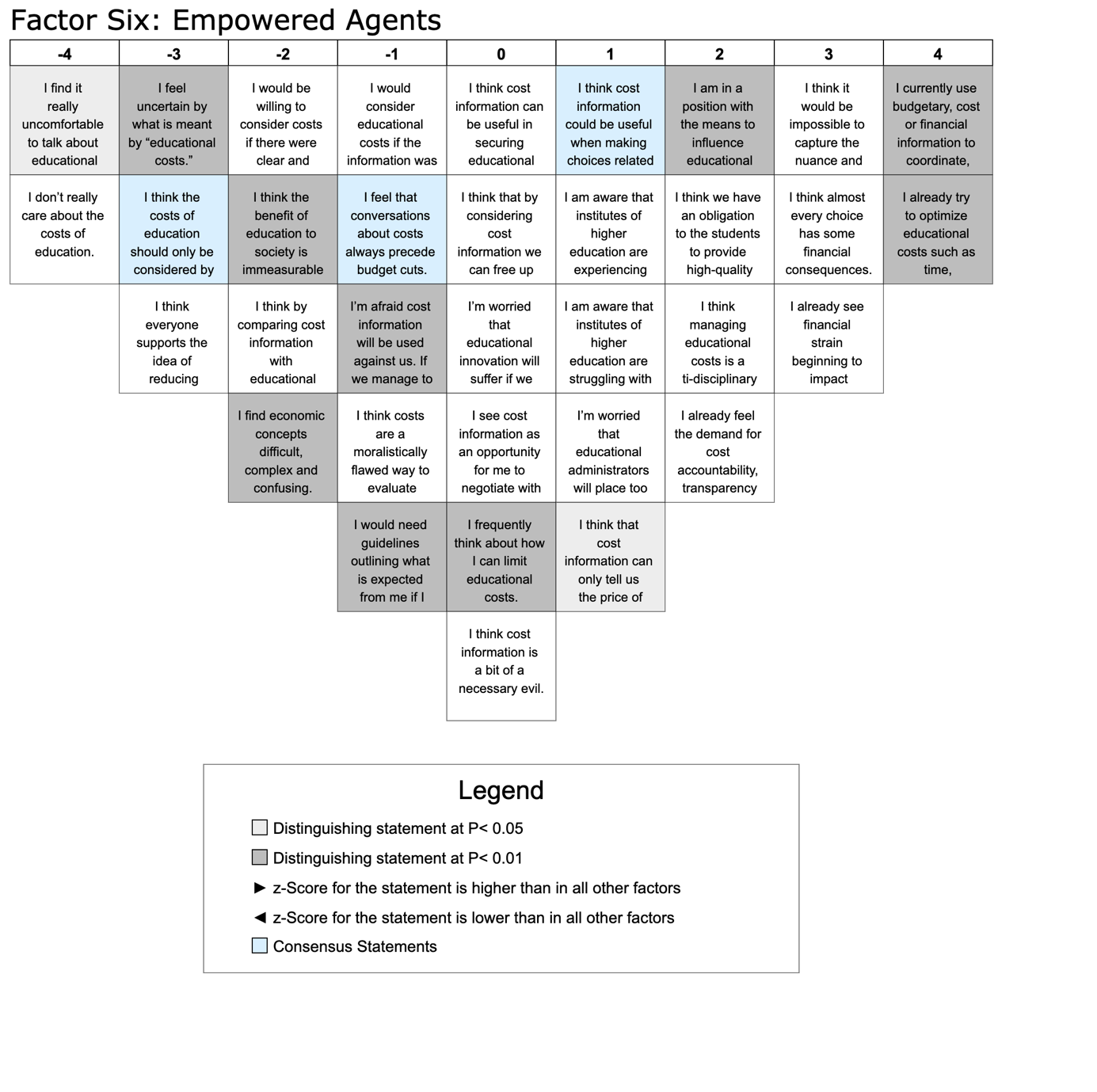
**

**5. Crib Sheets**

|  | **F1** | **Con/Dist** | **F2** | **F4** | **F6** |
| --- | --- | --- | --- | --- | --- |
| **Highest Ranked Statements** |  |  |  |  |  |
| I think we have an obligation to the students to provide high-quality education at a reasonable cost. | 4 |  | 3 | 4 | 2 |
| I am aware that institutes of higher education are struggling with personnel shortages. | 4 | D* | -1 | 1 | 1 |
| **Positive Statements Ranked Higher in Factor 1 Array than in Other Factor Arrays** |  |  |  |  |  |
| I’m worried that educational administrators will place too much emphasis on educational costs and not enough emphasis on educational outcomes or institutional objectives. | 3 |  | 2 | -1 | 1 |
| I already see financial strain beginning to impact educational activities around me. | 3 |  | -2 | -2 | 3 |
| I am aware that institutes of higher education are experiencing budget shortfalls. | 2 |  | -1 | 1 | 1 |
| I would be willing to consider costs if there were clear and transparent policies detailing how, when, and why cost information would be applied in decision-making. | 2 |  | 0 | 2 | -2 |
| I already feel the demand for cost accountability, transparency and efficiency in education. | 2 |  | -4 | 0 | 2 |
| I’m afraid cost information will be used against us. If we manage to save money one year, we’ll end up receiving less money the next year. | 1 |  | 1 | 0 | -1 |
| I feel uncertain by what is meant by “educational costs.” | 1 |  | -1 | 0 | -3 |
| **Negative Statements Ranked Lower in Factor 1 Array than in Other Factor Arrays** |  |  |  |  |  |
| I think cost information can be useful in securing educational funding. | 0 |  | 0 | 3 | 0 |
| I think that by considering cost information we can free up under-utilized resources for other uses. | -1 | D* | 1 | 2 | 0 |
| I feel that conversations about costs always precede budget cuts. | -1 | C | 0 | -1 | -1 |
| I’m worried that educational innovation will suffer if we start talking about costs. | -2 | D | 2 | -1 | 0 |
| I am in a position with the means to influence educational costs. | -3 |  | -3 | -3 | 2 |
| I think everyone supports the idea of reducing educational costs. | -3 |  | -1 | -2 | -3 |
| **Lowest Ranked Statements** |  |  |  |  |  |
| I think the costs of education should only be considered by economic, accounting, or financial experts and educational administrators | -4 | C | -2 | -4 | -3 |
| I think the benefit of education to society is immeasurable and worth whatever it costs. | -4 | D* | 2 | 1 | -2 |

**Factor 2: Unaware Doubters**

|  | **F2** | **Con/Dist** | **F1** | **F4** | **F6** |
| --- | --- | --- | --- | --- | --- |
| **Highest Ranked Statements** |  |  |  |  |  |
| I think it would be impossible to capture the nuance and complexity of education in terms of costs alone. | 4 |  | 2 | 1 | 3 |
| I think almost every choice has some financial consequences. | 4 |  | 1 | 3 | 3 |
| **Positive Statements Ranked Higher in Factor 2 Array than in Other Factor Arrays** |  |  |  |  |  |
| I think that cost information can only tell us the price of something, not its value or worth. | 3 | D* | 0 | -1 | 1 |
| I’m worried that educational innovation will suffer if we start talking about costs. | 2 | D* | -2 | -1 | 0 |
| I think costs are a moralistically flawed way to evaluate educational choices. | 2 | D* | -2 | -4 | -1 |
| I think the benefit of education to society is immeasurable and worth whatever it costs. | 2 |  | -4 | 1 | -2 |
| I think cost information is a bit of a necessary evil. | 1 |  | 0 | -1 | 0 |
| I’m afraid cost information will be used against us. If we manage to save money one year, we’ll end up receiving less money the next year. | 1 |  | 1 | 0 | -1 |
| I find economic concepts difficult, complex and confusing. | 1 |  | 0 | 0 | -2 |
| I don’t really care about the costs of education. | 0 | D* | -3 | -3 | -4 |
| I feel that conversations about costs always precede budget cuts. | 0 | C | -1 | -1 | -1 |
| **Negative Statements Ranked Lower in Factor 2 Array than in Other Factor Arrays** |  |  |  |  |  |
| I think cost information could be useful when making choices related to high-cost, high-volume and high-priority educational objectives. | 0 | C | 1 | 2 | 1 |
| I think cost information can be useful in securing educational funding. | 0 |  | 0 | 3 | 0 |
| I am aware that institutes of higher education are experiencing budget shortfalls. | -1 | D | 2 | 1 | 1 |
| I am aware that institutes of higher education are struggling with personnel shortages. | -1 | D* | 4 | 1 | 1 |
| I think by comparing cost information with educational outcomes we can improve educational quality. | -2 |  | -1 | 2 | -2 |
| I see cost information as an opportunity for me to negotiate with others in a persuasive manner. | -2 |  | -1 | 0 | 0 |
| I already see financial strain beginning to impact educational activities around me. | -2 |  | 3 | -2 | 3 |
| I am in a position with the means to influence educational costs. | -3 |  | -3 | -3 | 2 |
| I already try to optimize educational costs such as time, personnel, materials, equipment and facilities as efficiently and effectively as possible. | -3 | D* | 1 | 0 | 4 |
| I frequently think about how I can limit educational costs. | -3 |  | -2 | -2 | 0 |
| **Lowest Ranked Statements** |  |  |  |  |  |
| I already feel the demand for cost accountability, transparency and efficiency in education. | -4 | D* | 2 | 0 | 2 |
| I currently use budgetary, cost or financial information to coordinate, manage and execute educational responsibilities. | -4 | D* | -2 | -2 | 4 |

**Factor 4: Pragmatic Supporters**

|  | **F4** | **Con/Dist** | **F1** | **F2** | **F6** |
| --- | --- | --- | --- | --- | --- |
| **Highest Ranked Statements** |  |  |  |  |  |
| I think we have an obligation to the students to provide high-quality education at a reasonable cost. | 4 | D | 4 | 3 | 2 |
| I think managing educational costs is a multi-disciplinary effort. | 4 |  | 3 | 3 | 2 |
| **Positive Statements Ranked Higher in Factor 3 Array than in Other Factor Arrays** |  |  |  |  |  |
| I would need guidelines outlining what is expected from me if I had to consider the educational costs associated with my roles. | 3 | D | 0 | 1 | -1 |
| I think cost information can be useful in securing educational funding. | 3 | D* | 0 | 0 | 0 |
| I think cost information could be useful when making choices related to high-cost, high-volume and high-priority educational objectives. | 2 | C | 1 | 0 | 1 |
| I would be willing to consider costs if there were clear and transparent policies detailing how, when, and why cost information would be applied in decision-making. | 2 |  | 2 | 0 | -2 |
| I think by comparing cost information with educational outcomes we can improve educational quality. | 2 | D* | -1 | -2 | -2 |
| I think that by considering cost information we can free up under-utilized resources for other uses. | 2 |  | -1 | 1 | 0 |
| I would consider educational costs if the information was presented to me in summary reports. | 1 |  | 0 | 0 | -1 |
| I see cost information as an opportunity for me to negotiate with others in a persuasive manner. | 0 |  | -1 | -2 | 0 |
| **Negative Statements Ranked Lower in Factor 3 Array than in Other Factor Arrays** |  |  |  |  |  |
| I’m worried that educational administrators will place too much emphasis on educational costs and not enough emphasis on educational outcomes or institutional objectives. | -1 | D* | 3 | 2 | 1 |
| I think cost information is a bit of a necessary evil. | -1 |  | 0 | 1 | 0 |
| I feel that conversations about costs always precede budget cuts. | -1 | C | -1 | 0 | -1 |
| I think that cost information can only tell us the price of something, not its value or worth. | -1 |  | 0 | 3 | 1 |
| I already see financial strain beginning to impact educational activities around me. | -2 |  | 3 | -2 | 3 |
| I am in a position with the means to influence educational costs. | -3 |  | -3 | -3 | 2 |
| **Lowest Ranked Statements** |  |  |  |  |  |
| I think the costs of education should only be considered by economic, accounting, or financial experts and educational administrators | -4 | C | -4 | -2 | -3 |
| I think costs are a moralistically flawed way to evaluate educational choices. | -4 | D* | -2 | 2 | -1 |

**Factor 6: Empowered Agents**

|  | **F6** | **Con/Dist** | **F1** | **F2** | **F6** |
| --- | --- | --- | --- | --- | --- |
| **Highest Ranked Statements** |  |  |  |  |  |
| I currently use budgetary, cost or financial information to coordinate, manage and execute educational responsibilities. | 4 | D* | -2 | -4 | -2 |
| I already try to optimize educational costs such as time, personnel, materials, equipment and facilities as efficiently and effectively as possible. | 4 | D* | 1 | -3 | 0 |
| **Positive Statements Ranked Higher in Factor 4 Array than in Other Factor Arrays** |  |  |  |  |  |
| I already see financial strain beginning to impact educational activities around me. | 3 |  | 3 | -2 | -2 |
| I am in a position with the means to influence educational costs. | 2 | D* | -3 | -3 | -3 |
| I already feel the demand for cost accountability, transparency and efficiency in education. | 2 |  | 2 | -4 | 0 |
| I see cost information as an opportunity for me to negotiate with others in a persuasive manner. | 0 |  | -1 | -2 | 0 |
| I frequently think about how I can limit educational costs. | 0 | D* | -2 | -3 | -2 |
| **Negative Statements Ranked Lower in Factor 4 Array than in Other Factor Arrays** |  |  |  |  |  |
| I think cost information can be useful in securing educational funding. | 0 |  | 0 | 0 | 3 |
| I would consider educational costs if the information was presented to me in summary reports. | -1 |  | 0 | 0 | 1 |
| I feel that conversations about costs always precede budget cuts. | -1 | C | -1 | 0 | -1 |
| I’m afraid cost information will be used against us. If we manage to save money one year, we’ll end up receiving less money the next year. | -1 | D* | 1 | 1 | 0 |
| I would need guidelines outlining what is expected from me if I had to consider the educational costs associated with my roles. | -1 | D* | 0 | 1 | 3 |
| I would be willing to consider costs if there were clear and transparent policies detailing how, when, and why cost information would be applied in decision-making. | -2 |  | 2 | 0 | 2 |
| I think by comparing cost information with educational outcomes we can improve educational quality. | -2 |  | -1 | -2 | 2 |
| I find economic concepts difficult, complex and confusing. | -2 | D* | 0 | 1 | 0 |
| I feel uncertain by what is meant by “educational costs.” | -3 | D* | 1 | -1 | 0 |
| I think everyone supports the idea of reducing educational costs. | -3 |  | -3 | -1 | -2 |
| **Lowest Ranked Statements** |  |  |  |  |  |
| I find it really uncomfortable to talk about educational costs. | -4 | D | -1 | -1 | -3 |
| I don’t really care about the costs of education. | -4 |  | -3 | 0 | -3 |

**Supplementary file 6. Distinguishing Statements for Each Factor**

**Factor 1: Cautious Realists**

|  | **F1** | **F1** | **F1** | **F2** | **F2** | **F2** | **F4** | **F4** | **F4** | **F6** | **F6** | **F6** |
| --- | --- | --- | --- | --- | --- | --- | --- | --- | --- | --- | --- | --- |
| **Statement** | Q-SV | Z-score | Signif | Q-SV | Z-score | Signif | Q-SV | Z-score | Signifi | Q-SV | Z-score | Signifi |
| I am aware that institutes of higher education are struggling with personnel shortages. | 4 | 1,43 | ** | -1 | -0,332 | - | 1 | 0,599 | - | 1 | 0,706 | - |
| I think almost every choice has some financial consequences. | 1 | 0,42 | ** | 4 | 1,723 | - | 3 | 1,541 | - | 3 | 1,204 | - |
| I think that by considering cost information we can free up under-utilized resources for other uses. | -1 | -0,56 | ** | 1 | 0,417 | - | 2 | 0,602 | - | 0 | 0,217 | - |
| I’m worried that educational innovation will suffer if we start talking about costs. | -2 | -0,9 | * | 2 | 1,04 | - | -1 | -0,335 | - | 0 | 0,02 | - |
| I think the benefit of education to society is immeasurable and worth whatever it costs. | -4 | -1,84 | ** | 2 | 0,765 | - | 1 | 0,452 | - | -2 | -0,687 | - |

Q-SV represents the q-sort value corresponding to the placement of statement on the q-grid, *significance (p<0.05), **significance (p<0.01)

**Factor 2: Unaware Doubters**

|  | **F1** | **F1** | **F1** | **F2** | **F2** | **F2** | **F4** | **F4** | **F4** | **F6** | **F6** | **F6** |
| --- | --- | --- | --- | --- | --- | --- | --- | --- | --- | --- | --- | --- |
| **Statement** | Q-SV | Z-score | Signif | Q-SV | Z-score | Signif | Q-SV | Z-score | Signif | Q-SV | Z-score | Signif |
| I think that cost information can only tell us the price of something, not its value or worth. | 0 | -0,17 | - | 3 | 1,49 | * | -1 | -0,569 | - | 1 | 0,365 | - |
| I’m worried that educational innovation will suffer if we start talking about costs. | -2 | -0,9 | - | 2 | 1,04 | ** | -1 | -0,335 | - | 0 | 0,02 | - |
| I think costs are a moralistically flawed way to evaluate educational choices. | -2 | -0,79 | - | 2 | 0,8 | ** | -4 | -1,742 | - | -1 | -0,468 | - |
| I don’t really care about the costs of education. | -3 | -1,57 | - | 0 | 0,09 | ** | -3 | -1,647 | - | -4 | -1,972 | - |
| I am aware that institutes of higher education are experiencing budget shortfalls. | 2 | 1,13 | - | -1 | -0,3 | * | 1 | 0,346 | - | 1 | 0,861 | - |
| I am aware that institutes of higher education are struggling with personnel shortages. | 4 | 1,43 | - | -1 | -0,33 | ** | 1 | 0,599 | - | 1 | 0,706 | - |
| I already try to optimize educational costs such as time, personnel, materials, equipment and facilities as efficiently and effectively as possible. | 1 | 0,52 | - | -3 | -1,3 | ** | 0 | 0,117 | - | 4 | 1,284 | - |
| I already feel the demand for cost accountability, transparency and efficiency in education. | 2 | 0,73 | - | -4 | -1,49 | ** | 0 | 0,037 | - | 2 | 0,891 | - |
| I currently use budgetary, cost or financial information to coordinate, manage and execute educational responsibilities. | -2 | -0,84 | - | -4 | -1,87 | ** | -2 | -0,836 | - | 4 | 1,611 | - |

Q-SV represents the q-sort value corresponding to the placement of statement on the q-grid,*significance (p<0.05), **significance (p<0.01)

**Factor 4: Pragmatic Supporters**

|  | **F1** | **F1** | **F1** | **F2** | **F2** | **F2** | **F4** | **F4** | **F4** | **F6** | **F6** | **F6** |
| --- | --- | --- | --- | --- | --- | --- | --- | --- | --- | --- | --- | --- |
| **Statement** | Q-SV | Z-score | Signif | Q-SV | Z-score | Signif | Q-SV | Z-score | Signif | Q-SV | Z-score | Signif |
| I think we have an obligation to the students to provide high-quality education at a reasonable cost. | 4 | 1,59 | - | 3 | 1,25 | - | 4 | 2,22 | * | 2 | 1,035 | - |
| I would need guidelines outlining what is expected from me if I had to consider the educational costs associated with my roles. | 0 | 0,23 | - | 1 | 0,57 | - | 3 | 1,27 | * | -1 | -0,499 | - |
| I think cost information can be useful in securing educational funding. | 0 | 0,31 | - | 0 | 0,12 | - | 3 | 1,26 | ** | 0 | 0,353 | - |
| I think by comparing cost information with educational outcomes we can improve educational quality. | -1 | -0,76 | - | -2 | -0,92 | - | 2 | 0,75 | ** | -2 | -0,93 | - |
| I think it would be impossible to capture the nuance and complexity of education in terms of costs alone. | 2 | 1,36 | - | 4 | 1,79 | - | 1 | 0,56 | * | 3 | 1,235 | - |
| I already feel the demand for cost accountability, transparency and efficiency in education. | 2 | 0,73 | - | -4 | -1,49 | - | 0 | 0,04 | ** | 2 | 0,891 | - |
| I’m worried that educational administrators will place too much emphasis on educational costs and not enough emphasis on educational outcomes or institutional objectives. | 3 | 1,43 | - | 2 | 1,07 | - | -1 | -0,41 | ** | 1 | 0,537 | - |
| I think costs are a moralistically flawed way to evaluate educational choices. | -2 | -0,79 | - | 2 | 0,8 | - | -4 | -1,74 | ** | -1 | -0,468 | - |

Q-SV represents the q-sort value corresponding to the placement of statement on the q-grid, *significance (p<0.05), **significance (p<0.01)

**Factor 6: Empowered Agents**

|  | **F1** | **F1** | **F1** | **F2** | **F2** | **F2** | **F4** | **F4** | **F4** | **F6** | **F6** | **F6** |
| --- | --- | --- | --- | --- | --- | --- | --- | --- | --- | --- | --- | --- |
| **Statement** | Q-SV | Z-score | Signif | Q-SV | Z-score | Signif | Q-SV | Z-score | Signif | Q-SV | Z-score | Signif |
| I currently use budgetary, cost or financial information to coordinate, manage and execute educational responsibilities. | -2 | -0,84 | - | -4 | -1,87 | - | -2 | -0,84 | - | 4 | 1,61 | ** |
| I already try to optimize educational costs such as time, personnel, materials, equipment and facilities as efficiently and effectively as possible. | 1 | 0,52 | - | -3 | -1,3 | - | 0 | 0,12 | - | 4 | 1,28 | ** |
| I am in a position with the means to influence educational costs. | -3 | -1,05 | - | -3 | -1,25 | - | -3 | -1,25 | - | 2 | 1,08 | ** |
| I think that cost information can only tell us the price of something, not its value or worth. | 0 | -0,17 | - | 3 | 1,49 | - | -1 | -0,57 | - | 1 | 0,37 | * |
| I frequently think about how I can limit educational costs. | -2 | -1,01 | - | -3 | -1,45 | - | -2 | -0,88 | - | 0 | -0,18 | ** |
| I’m afraid cost information will be used against us. If we manage to save money one year, we’ll end up receiving less money the next year. | 1 | 0,6 | - | 1 | 0,66 | - | 0 | 0,27 | - | -1 | -0,43 | ** |
| I would need guidelines outlining what is expected from me if I had to consider the educational costs associated with my roles. | 0 | 0,23 | - | 1 | 0,57 | - | 3 | 1,27 | - | -1 | -0,5 | ** |
| I think the benefit of education to society is immeasurable and worth whatever it costs. | -4 | -1,84 | - | 2 | 0,77 | - | 1 | 0,45 | - | -2 | -0,69 | ** |
| I find economic concepts difficult, complex and confusing. | 0 | -0,1 | - | 1 | 0,35 | - | 0 | -0,22 | - | -2 | -1,21 | ** |
| I feel uncertain by what is meant by “educational costs.” | 1 | 0,43 | - | -1 | -0,24 | - | 0 | 0,11 | - | -3 | -1,35 | ** |
| I find it really uncomfortable to talk about educational costs. | -1 | -0,53 | - | -1 | -0,65 | - | -3 | -1,2 | - | -4 | -1,84 | * |

Q-SV represents the q-sort value corresponding to the placement of statement on the q-grid, *significance (p<0.05), **significance (p<0.01)

**Supplementary file 7. Consensus Statements Between All Factors**

|  | **F1** | **F1** | **F2** | **F2** | **F4** | **F4** | **F6** | **F6** |
| --- | --- | --- | --- | --- | --- | --- | --- | --- |
| **Statement** | Q-SV | Z-score | Q-SV | Z-score | Q-SV | Z-score | Q-SV | Z-score |
| I think cost information could be useful when making choices related to high-cost, high-volume and high-priority educational objectives. | 1 | 0,36 | 0 | 0,15 | 2 | 0,88 | 1 | 0,86 |
| I think the costs of education should only be considered by economic, accounting, or financial experts and educational administrators | -4 | -1,68 | -2 | -0,95 | -4 | -1,71 | -3 | -1,466 |
| I feel that conversations about costs always precede budget cuts. | -1 | -0,75 | 0 | 0 | -1 | -0,53 | -1 | -0,401 |

Q-SV represents the q-sort value corresponding to the placement of statement on the q-grid

**Supplementary file 8. Sensitivity Analysis of Factor Loading Cut-Off Values on Brown CFA 6-Factor Solution**

Determination of which q-sorts to retain in each factor array was made after selection of the Brown CFA 6-Factor solution. In the Brown CFA 6-Factor solution, all q-sorts loaded significantly on a factor (p<0.001) and could be retained in the model. However, the degree to which a q-sort contributes to explained variance is derived from the factor loading value, which represents how closely correlated each q-sort is with a factor (+1 indicating perfect correlation, 0 indicating no correlation, and -1 indicating perfect opposition). In Varimax rotation, q-sorts are associated by default to the factor with which they demonstrate the highest correlation, regardless of the level of correlation or the extent of variance explained. Within the Brown CFA 6-Factor solution, significant factor loading values ranged from +0.84 to +0.35, indicating some q-sorts were less strongly aligned and contributed less to explained variance than others. Therefore, a sensitivity analysis of factor-loading cut-off values was conducted to observe the effect on solution clarity, interpretability and reliability.

Upon completion of analysis, a factor-loading cut-off value of 0.50 was selected as it allowed for additional patterns of consensus to emerge, increased the percentage of explained variance, maintained a high level of reliability, while minimizing the number of q-sorts excluded from analysis and resulting in only minor alterations to typologies.

**Sensitivity Analysis of Factor Loading Cut-Off Values**

| Factor loading cut-off Value | None^1^ | 0.40 | 0.50 | 0.55 |
| --- | --- | --- | --- | --- |
| Q-sorts significantly loading on a factor | 29 | 29 | 29 | 29 |
| Q-sorts loading on or above the cut-off value | 29 | 28 | 25 | 19 |
| Q-sorts per factor  (F1-F2-F3-F4) | 8-4-10-7 | 8-4-9-7 | 8-3-8-6 | 5-3-5-6 |
| Variance Explained (%) | 56 | 58 | 62 | 62 |
| Variance Explained (%) per Factor (F1-F2-F3-F4) | 15-11-14-16 | 15-11-15-17 | 16-12-16-18 | 14-14-14-20 |
| Composite Reliability of Factors  (F1-F2-F3-F4) | 0.97-0.94-0.98-0.97 | 0.97-0.92-0.97-0.97 | 0.97-0.92-0.97-0.96 | 0.95-0.92-0.96-0.96 |
| Correlation between factors | Moderate | No noticeable change | No noticeable change | Slightly reduced |
| Demographic Profile | Distinct profiles for factors 2 and 4 | No noticeable change | Slightly more distinct for all factors with minimal participants excluded. | Still more distinct for each factor. However, more experienced educators are dropped from Factor 4. Typology remains relatively unchanged |
| Interpretation | Same as Brown CFA 6 Factor | No noticeable change | Additional consensus statement emerges, revealing a new shared pattern of thinking | Consensus statements change slightly, but emphasis and interpretation remain the same |

^1^ Q-sorts assigned to the factor upon which it loaded highest (was most correlated)

**Supplementary file 9. Correlation of Factors**

|  | **Factor 1** | **Factor 2** | **Factor 4** | **Factor 6** |
| --- | --- | --- | --- | --- |
| **Factor 1** | 1 | 0.2928 | 0.576 | 0.5636 |
| **Factor 2** | 0.2928 | 1 | 0.429 | 0.0718 |
| **Factor 4** | 0.576 | 0.429 | 1 | 0.3843 |
| **Factor 6** | 0.5636 | 0.0718 | 0.3843 | 1 |
